# Weight-Related Teasing of Adolescents Who Are Primarily Obese: Roles of Sociocultural Attitudes Towards Appearance and Physical Activity Self-Efficacy

**DOI:** 10.3390/ijerph16091540

**Published:** 2019-04-30

**Authors:** Carolyn E. Ievers-Landis, Carly Dykstra, Naveen Uli, Mary Ann O’Riordan

**Affiliations:** 1 Division of Developmental/Behavioral Pediatrics & Psychology, Department of Pediatrics, Rainbow Babies & Children’s Hospital, UH Hospitals Cleveland Medical Center, Cleveland, OH 44106-6038, USA; 2 Department of Pediatrics, Rainbow Babies & Children’s Hospital, UH Hospitals Cleveland Medical Center, Cleveland, OH 44106, USA; carly.dykstra@uhhospitals.org (C.D.); MaryAnn.O’Riordan@UHhospitals.org (M.A.O.); 3 Division of Pediatric Endocrinology & Metabolism, Department of Pediatrics, Rainbow Babies & Children’s Hospital, UH Hospitals Cleveland Medical Center, Cleveland, OH 44106, USA; Naveen.uli1@uhhospitals.org

**Keywords:** adolescent, obesity, teasing, weight-related teasing, appearance, body image, physical activity self-efficacy, sociocultural attitudes towards appearance

## Abstract

Adolescents who are obese are at risk for being teased about their appearance with the concomitant negative psychological sequelae. Identifying modifiable variables associated with teasing could inform pediatric weight-management interventions. Characterizing society’s role in the victimization of these at-risk individuals could guide anti-bullying programs for schools and broader public health efforts. This study aims to examine novel societal and cognitive factors associated with weight-related teasing frequency. Participants were adolescents (*N* = 334) being evaluated for a hospital-affiliated weight-management program. The outcome was perceived weight-related teasing frequency. Predictors were sociocultural awareness and internalization of appearance-related attitudes, physical activity self-efficacy, and psychological functioning. Multivariate regressions controlled for demographics and body mass index (BMI) z-scores with separate regressions testing interactions of BMI z-scores with all predictors. In adjusted analyses, higher physical activity self-efficacy and fewer depressive symptoms related to lower teasing frequency. Interactions indicated that less awareness/internalization of sociocultural attitudes towards appearance, more positive body image, and higher self-esteem related to lower teasing frequency regardless of BMI. Targeted interventions and public health campaigns should be developed and tested for adolescents that improve body image with promotion of diverse views about attractiveness, bolster confidence in overcoming physical activity barriers, and identify and treat mood symptoms.

## 1. Introduction

Weight stigmatization is prevalent towards those in our society who are overweight or obese, with research documenting discrimination over the past half a century and more focused attention in the field of obesity over the past 15 years [1]. This stigmatization affects adults over a broad scope of their lives, including employment, health care, education, media coverage, and their personal relationships [1]. Even adolescents experience weight-related teasing, with estimates of about 18% for males and 23–28% for females from a sample of 2287 participants in Project EAT-III [2]. Higher percentages were reported among the subgroup of adolescents who were overweight or obese with ranges of approximately 29–37% for males and 28–40% for females [2]. An assessment of weight-based victimization was conducted in a sample of adolescents enrolled in weight loss camps (*N* = 361) with 24% being overweight, 40% being obese, and the remainder being normal weight due to having experienced significant weight loss and returned to camp for support with maintenance. In this unique sample as many as 78% reported being teased about their weight over the past year with 36% endorsing being the target of bullying for five years [3]. These adolescents reported being teased mostly by peers (92%) and friends (70%), but they also indicated that adults were involved including parents (37%) and teachers (27%), with the most common being physical education teachers/coaches (42%) [3]. Adolescents who are obese are at an even greater risk of being teased compared to their normal-weight peers [4,5,6], which might be one factor explaining the differences in psychological well-being between normal weight youth versus those who are obese. 

Peer victimization in childhood and adolescence has been found to be associated with depressive symptoms, poor self-esteem, social anxiety, loneliness, poor academic performance, and disordered eating behaviors [4,7,8]. Weight-based teasing in adolescence predicts eating to cope with negative affect and obesity, and the potential ramifications of this type of teasing extend into adulthood for both females and males [9]. Similar research findings exist for adolescents who are already overweight or obese. For example, in a sample of 343 adolescents in Grades 6 through 8 who were overweight or obese, self-esteem and body satisfaction served as mediators in the relationship between teasing and depressive symptoms [10]. This finding led to the conclusion that more positive self-evaluations may protect against the negative influence of teasing on mood [10]. Within a sample more similar to the current study’s, among 93 children and adolescents who were overweight (7.5%) or obese (92.5%) and seeking treatment, child-reported weight-related teasing was found to relate to lower health-related quality of life across three time periods over 15 months [11]. Rancourt and colleagues followed 119 adolescents who were obese and participated in a weight control intervention over four time points (baseline, post-intervention, 12 months and 24 months post-randomization) and found that decreases in percent overweight were prospectively associated with subsequent declines in weight-related teasing [12]. 

The specific factors other than body mass index (BMI) that explain the frequency of weight-related teasing are still not clear despite research efforts in this area. For example, in a multicenter study of school-aged children, the relationship between weight-related teasing and BMI was found to be independent of sex, race/ethnicity, family socioeconomic status (SES), school demographic profile, social skills, and academic achievement [13]. Additionally, a finding from a large study of adolescents (*N* = 4746) was that weight-related teasing did not differ by racial/ethnic group [6]. 

To add to the literature in this area, the present study’s focus is on two novel predictors of weight-related teasing that may serve as modifiable targets for intervention regardless of BMI in a high-risk population of adolescents who are primarily obese and seeking treatment. These are awareness and internalization of sociocultural attitudes about appearance and physical activity self-efficacy. How much society plays a role in the phenomenon of being teased for one’s appearance has been investigated for a different societal construct—weight bias internalization—for this particularly at-risk population of adolescents who are overweight or obese. A recent study examined the relationship of weight bias internalization among 148 adolescents seeking weight loss, with this construct defined as the process of self-stigma of being aware of the negative stereotypes about one’s identity, agreeing with these stereotypes, applying them to oneself, and feeling devalued [14]. Findings were that internalized weight bias was significantly related to weight-based teasing from peers [14]. More research is needed in the area of weight-related teasing regarding how both the awareness and internalization of more general societal attitudes toward attractiveness, not just weight bias, might influence adolescents who are obese and seeking treatment and their likelihood of being teased based upon their appearance. This question involves not only the adolescent’s awareness of how society defines attractiveness but also the internalization of this definition as it applies to the adolescent [15,16]. In other words, awareness applies to the degree to which the adolescent is cognizant of society’s attitudes toward attractiveness or beauty, and internalization refers to how much the adolescent directs these attitudes to himself or herself. Particularly as adolescents’ awareness and internalization of society’s attitudes towards appearance may be modified via psychological interventions or public health education efforts, examining whether society is playing a role in weight-related teasing is a worthy research endeavor. Research on this construct among female adolescents across the weight spectrum has found that girls in co-educational environments compared to single-sex schools had poorer self-esteem related to greater internalization of sociocultural attitudes toward appearance [17]. Similarly, for a sample of males (16.7% who were obese) and females (25% who were obese), pressure to be thin and muscular from peers was associated with more frequent emotional eating with internalization of appearance-related ideals serving as a mediator [18].

Another potential novel factor to examine as a predictor of weight-related teasing is cognitions regarding physical activity. Some research suggests that teasing from peers is associated with engagement in physical activity. Storch and colleagues found that among youth aged 8 to 18 years, peer victimization was negatively correlated with level of physical activity [8]. Similarly, a study of elementary school children found that weight-related teasing during physical activity was associated with lower levels of physical activity [19]. This relationship may be explained by reductions in physical activity self-efficacy, as self-efficacy has been found to be strongly associated with physical activity in youth [20]. Self-efficacy is the belief or personal cognition/perception regarding one’s own capability to succeed in a specific situation. Physical activity self-efficacy refers to the belief in one’s own capability to participate in physical activity and to choose to be active even when there are barriers. In adolescents, the relationship between female sex, higher weight status and lower physical activity self-efficacy has been documented, but its relationship to weight-related teasing has not yet been studied exclusively among children or adolescents who are overweight or obese [21,22]. In a sample of 321 primarily normal weight 5th–8th Grade children (only 9% were overweight, including four who were obese), Losekam and colleagues studied a similar objective with self-reported height and weight and physical activity measures [23]. Higher BMI percentile was associated with lower physical activity self-efficacy for boys but not for girls, and teasing was associated with lower physical activity self-efficacy for both boys and girls. To expand on this area of inquiry, studies should include an older age range of children with a larger proportion of those who are overweight or obese. Characterization of significant correlates of teasing in this population is essential for identifying treatment targets for pediatric weight-management programs to reduce teasing and its adverse psychosocial effects. 

In summary, the primary objective of the present study was to evaluate expanded models of correlates of weight-related teasing in a treatment-seeking sample of adolescents who were primarily obese, including a focus on awareness and internalization of society’s definitions of attractiveness and physical activity self-efficacy. Hypotheses were that a higher reported frequency of weight-related teasing among treatment-seeking adolescents who are overweight or obese would relate to higher BMI z-scores. For models testing the relationship of societal, cognitive, and psychological factors with weight-related teasing, hypotheses were that greater awareness and internalization of societal attitudes about attractiveness, lower physical activity self-efficacy, and worse psychological functioning would be significantly related to higher teasing frequency. Finally, hypotheses were that significant interactions of BMI z-scores and the societal/cognitive/psychological variables would indicate that BMI z-scores would relate to weight-related teasing frequency for those with a greater impact of society’s views as well as lower physical activity self-efficacy and psychological functioning. Conversely, significantly weaker or no relationship was expected between weight-related teasing and BMI z-scores for adolescents with less awareness/internalization of society’s attractiveness views, greater confidence in their ability to be physically active, and healthier psychological functioning.

## 2. Materials and Methods

### 2.1. Participants and Procedure

Participants were adolescents evaluated from approximately 2006–14 for a children’s hospital-affiliated outpatient weight-management program at a relatively large urban center that included several suburban locations for subsequent family-based group treatment sessions. The principal investigators of the clinical research program were two pediatric endocrinologists and a licensed clinical psychologist, and the staff included exercise physiologists, psychology assistants, and registered dieticians. This study was approved by the local institution’s Institutional Review Board. Prior to participation in the initial evaluation, parents or legal guardians and their children met with trained staff members to explain the clinical research aspect of the program. If they chose to participate in the research portion of the program, adults went through the informed consent process and signed a consent form, and adolescents signed assent forms. Children were given the opportunity to decline participation in the research portion of the program at any time while still receiving clinical services. Medical insurance was charged for the medical evaluation conducted by a pediatric endocrinologist, and all other aspects of the evaluation and the later group treatment or individual sessions were provided at no cost to families. Data were collected from adolescents and their parents/legal guardians during their initial outpatient evaluation clinic visit for those consenting to be part of the study. Participants’ height and weight were measured by staff. Exercise physiologists conducted the test of body composition. Participants and parents completed measures prior to or during the clinic visit; a subset of these are included in the present study. 

### 2.2. Measures

#### 2.2.1. Perception of Teasing Scale (POTS)

The outcome variable was perception of weight-related teasing from the Perception of Teasing Scale (POTS [24]). Adolescents completed the POTS to evaluate their perceived frequency of weight-related teasing. This 11-item measure assesses the extent to which adolescents experience teasing related to weight and competency, and only the 5-item weight-related teasing subscale was used in this study. Items include: “People made fun of you because you were heavy”, “People made jokes about you being heavy”, “People laughed at you for trying out for sports because you were heavy”, “People called you names like ‘Fatso’”, and “People pointed at you because you were overweight”. Ratings are made using a 5-point Likert scale from “Never” to “Very often”, with higher scores indicating more frequent teasing. A mean score was calculated for each participant. Initial validity gathered for this measure was that weight-related teasing correlated with measures of body image, eating disturbance, and self-esteem. Internal consistency and two-week test-retest reliabilities have been acceptable (r = 0.88) [24]. In a similar sample of treatment-seeking children and adolescents who were primarily obese, internal consistency of the weight-related teasing subscale at three time points was quite high (r’s = 0.90−0.91) [11]. Additionally, as evidence of the validity of the POTS for the current study, the weight-related teasing subscale in their sample was inversely associated with quality of life at all three time points [11].

#### 2.2.2. Demographics, Weight Status, and Body Composition

Parents completed a standard measure to gather demographic information, which for the present study included child age, sex, race (dichotomized as White versus Non-White), and parent education (dichotomized as high school education or greater than high school/higher education). Weight status was obtained via measurement of the participants’ height and weight. BMI was calculated, and BMI z-score was derived for age and gender on the basis of the charts published by the Centers for Disease Control in 2000 [25]. To be eligible for participation in the weight-management program, participants must have had a BMI above the 85th percentile for age and sex. A measure of body composition or adiposity was also included in the present study. Percent body fat, fat mass (kg), and lean mass (kg) were estimated using air displacement plethysmography with the BOD POD ^®^ Body Composition Tracking System, which is a computerized egg-like chamber [26]. The BOD POD uses the principle of whole-body densitometry to determine the amount of fat and lean tissue in the body. This technique is comparable to underwater weighing, but displacement of air is measured instead of water to determine body volume. The BOD POD test was conducted with participants at the evaluation visit. This test required a two-hour fast, and participants were instructed to not exercise three hours prior to the test. Participants wore minimal, skin-tight clothing (e.g., swimsuit, sports bra and biking shorts). Participants entered the BOD POD and sat still while breathing normally for two 60-second measures. This procedure took approximately 15 min. 

#### 2.2.3. Sociocultural Attitudes Towards Appearance Questionnaire (SATAQ) 

Two aspects of female-only participants’ perceptions about society’s attitudes towards appearance were assessed with the Sociocultural Attitudes Towards Appearance Questionnaire (SATAQ [15]). The SATAQ was originally designed and validated for females only and contains items that reflect appearance concerns that are inappropriate for use with male samples (e.g., pressure to look pretty) [16]. In 2015, Schaefer and colleagues reported on the development and validation of the SATAQ-4 that was modified to address appearance concerns more relevant to males; an underlying factor structure was found that was unique to males [16]. The original SATAQ used for the present study is a 14-item empirically developed measure with two subscales, awareness and internalization. Awareness contains 6 items that serve as an index of acknowledgement of societal appearance norms (e.g., “Most people believe that the thinner you are, the better you look”). The other subscale, Internalization, includes 8 items such as: “I tend to compare my body to people in magazines and on TV”. Higher mean scores reflect more awareness and internalization. Internal consistency was found to be adequate to high with an alpha of 0.71 for the Awareness subscale and 0.88 for the Internalization subscale [15]. Evidence for the validity of the SATAQ was obtained via factor analysis of the two distinct factors; both subscales are associated with multiple indicators of body image and eating disturbance [15].

#### 2.2.4. Physical Activity Self-Efficacy Scale (PASES)

Physical activity self-efficacy was measured using the Physical Activity Self-Efficacy Scale (PASES [27]). The PASES is an 8-item measure of participants’ perceptions of how confident they feel about their ability to make changes in their physical activity level despite potential barriers. The ratings are made using a 4-point Likert scale (1 = “Strongly agree” to 4 = “Strongly disagree”). An example item is, “I can be physically active during my free time on most days”. The mean score of the 8 items was used in this study, and lower scores indicate greater self-efficacy. Evidence of validity for this subscale as a unidimensional measure of self-efficacy for physical activity was gathered via confirmatory factor analysis when compared with the other dimensions of attitudes, subjective norms, and perceived behavioral control for physical activity [22]. Additional validity, from a sample of adolescents who were primarily obese in the same clinical research program as the current study, was that higher physical activity self-efficacy as measured by the PASES was associated with lower resting heart rate and more positive family functioning (higher cohesion, lower conflict) [28].

#### 2.2.5. Self-Image Questionnaire for Young Adolescents (SIQYA)

Body image was assessed by the Self-Image Questionnaire for Young Adolescents (SIQYA [29]) with the 11-item Body Image subscale. Higher scores indicate a more positive body image. An example of the five positively worded items is, “Most of the time, I am happy with the way I look”. An example of the six negatively worded items that are reverse scored is, “I am not satisfied with my weight”. The alpha coefficient for the Body Image subscale was high (i.e., high internal consistency of the items) [29]. Evidence for discriminant validity was provided via factor analysis, differentiating the Body Image subscale from the other eight subscales (e.g., Emotional Tone, Impulse Control); convergent validity evidence was gathered through the association of the Body Image subscale with other measures of self-image [29].

#### 2.2.6. Rosenberg Self-Esteem (RSE) Scale and Center for Epidemiological Studies-Depression Scale for Children (CES-DC)

Two additional psychological functioning measures included self-esteem and depressive symptomatology. Participant self-esteem was measured by the Rosenberg Self-Esteem (RSE) scale [30], a 10-item questionnaire with responses ranging from 1 (“Strongly agree”) to 5 (“Strongly disagree”) on a Likert scale. The sum of the 10 items was coded with lower scores equaling higher self-esteem. This scale has well-established predictive validity, high internal consistency, and test-retest reliability [30]. Participants’ mood states were assessed using the Center for Epidemiological Studies-Depression Scale for Children (CES-DC [31]). The CES-DC assesses depressive symptoms in non-psychiatric populations and is made up of 20 items scored on a 4-point scale (0 to 3). Participants report the frequency of affective, cognitive, and motivational symptoms of depressed mood experienced over the past week. The CES-DC is scored by summing the responses for the 20 items with higher scores reflecting greater severity. The CES-DC is validated for children and young adults between the ages of 6 and 23 years and has well-established reliability and validity for measuring depressive symptoms particularly between the ages of 12 to 18 [31]. Children diagnosed as having major depressive disorder or dysthymia are more likely to have elevated scores compared to those without these diagnoses [31].

### 2.3. Statistical Analyses

All analyses were done using SAS, v 9.3 (The SAS Institute, Cary, NC, USA). Spearman rank-order correlations or chi-square analyses were conducted between the outcome and all predictor variables. Predictors were selected demographics (age, race, sex, and parent education), weight status (BMI z-scores), body composition (BOD POD measures), awareness/internalization of societal attitudes toward appearance, physical activity self-efficacy, and psychological factors (body image, self-esteem, depressive symptomatology). Hierarchical regression models were run for each predictor variable separately, controlling first for demographics, then for BMI z-scores. Results are reported as the significance of the change in R^2^ after the addition of the variable of interest. Note that BMI was not included in any of the models with the BOD POD variables due to their high correlations. Finally, separate hierarchical regression models were conducted for each of the predictor variables with an interaction term of each factor by BMI z-score being entered in the last step. For the figures, predictor variables with significant interactions were classified as high or low based on values above and below the median, respectively. With our sample size (*N* = 334), an effect size of 0.21 can be detected with alpha = 0.05, 80% power. Effect sizes of 0.20 are considered small. Additionally, for the regression analyses, with three variables in the model with a correlation of 0.3, power was sufficient to detect a difference in R^2^ of 0.02 when adding a fourth variable. This does not change with correlations as low as 0.1. Therefore, our study was adequately powered for the analyses that were conducted. 

## 3. Results

### 3.1. Characteristics of the Participants

Data from 334 participants, ranging in age from 13 to 18 years with a mean of 14.59 years (SD = 1.38), were included in the present study. The majority of this sample was female (63.77%), and the race/ethnic makeup was approximately half White (50.63%) and half Non-White. For parental education, almost three-quarters (73.35%) reported having more than a high school education. The sample was primarily obese, and the BMI z-score mean was 2.10 (SD = 1.10). See Table 1 for properties of the major study variables, including the sample size, mean, standard deviation, Cronbach’s alpha (reliability/internal consistency), and the potential/actual ranges.

### 3.2. Bivariate Relationships of the Outcome with Other Variables

Spearman rank-order correlations were conducted between the outcome variable and all continuous predictors, and chi-square analyses were conducted for nominal variables (see Table 2). Table 2 provides statistics regarding the bivariate analyses of all of the predictors with frequency of weight-related teasing. The only demographic factor significantly related to weight-related teasing frequency was parental education. Adolescents who had parents with a high school education or less reported significantly more teasing (M = 2.40; SD = 1.03) compared to those who had parents with more than a high school degree (M = 2.00; SD = 1.25) (*p* = 0.008). Sex, race, and age were not significantly associated with teasing. As predicted, significant associations were found for higher frequency of weight-related teasing and BMI z-scores (*ρ* = 0.31, *p* < 0.001) as well as the following BOD POD variables: body fat percentage (*ρ* = 0.28, *p* < 0.001), fat mass (kg) (*ρ* = 0.29, *p* < 0.001), and lean mass (kg) (*ρ* = 0.15, *p* < 0.02). Both societal variables were significantly associated with greater weight-related teasing: higher sociocultural attitude awareness (*ρ* = 0.27; *p* < 0.001) and internalization (*ρ* = 0.36; *p* < 0.001). Lower physical activity self-efficacy was significantly related to greater weight-related teasing (*ρ* = 0.22, *p* < 0.001). Less positive body image was also related to higher teasing frequency (*ρ* = −0.45; *p* < 0.001). For the other psychological functioning variables, the hypothesized significant associations were found for more frequent weight-related teasing with lower self-esteem (*ρ* = 0.42; *p* < 0.001) and greater depressive symptoms (*ρ* = 0.42; *p* < 0.001).

### 3.3. Hierarchical Multiple Regression Analyses

Refer to Table 3 for regression analysis models predicting frequency of weight-related teasing. In the first hierarchical multiple regression, higher BMI z-scores were significantly related to more frequent weight-related teasing (*p* < 0.001) after controlling for demographics. Four separate multiple regression analyses yielded significance for two measures of adiposity, physical activity self-efficacy, and a psychological functioning variable after controlling for BMI z-scores (except for the BOD POD variable models) and demographics. Adolescents who had a higher body fat percentage (*p* < 0.001) and parents with less education (*p* < 0.05) reported more frequent weight-related teasing. Additionally, having parents with less education (*p* < 0.05) and having higher fat mass (*p* < 0.002) was related to more frequent weight-related teasing. Finally, being White (*p* < 0.05), female (*p* < 0.02), and having a higher BMI z-score (*p* < 0.001) and lower physical activity self-efficacy (*p* < 0.002) related to more frequent teasing. A multiple regression for the psychological functioning variables indicated that having a higher BMI z-score (*p* < 0.001) and greater depressive symptoms (*p* < 0.001) related to experiencing more frequent weight-related teasing.

### 3.4. Multiple Regression Analyses Testing Interactions

**Figure 1:**
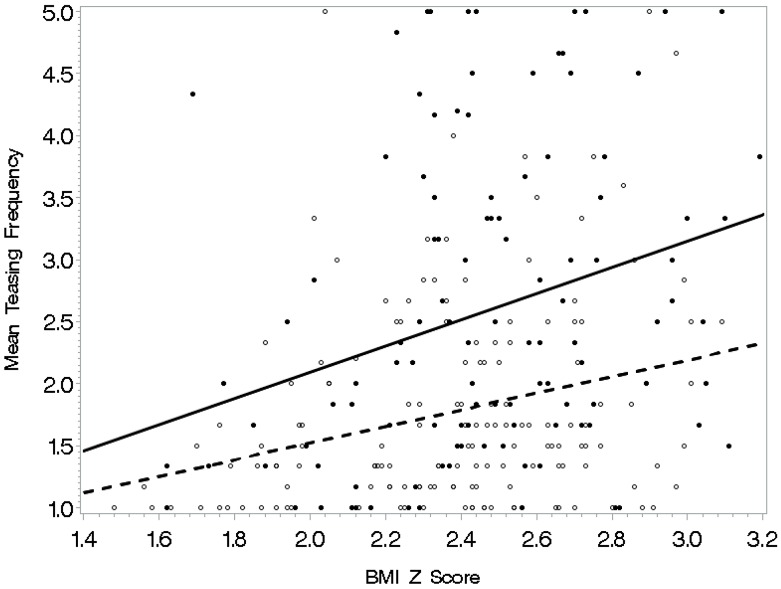
Body image as a moderator: BMI z-scores related less to weight-related teasing frequency for adolescents with a more positive body image (dashed line) compared to those with a more negative body image (solid line).

**Figure 2:**
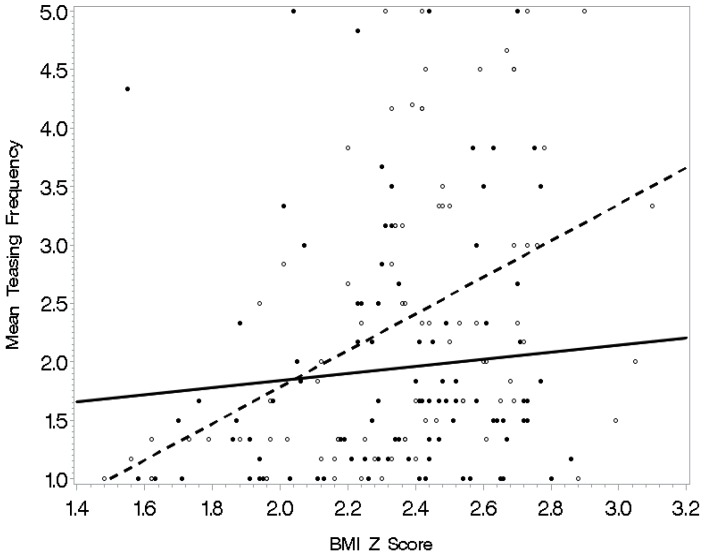
Awareness of society’s attitudes toward appearance as a moderator: A minimal relationship existed between BMI z-scores and teasing frequency for those adolescents who were less aware of society’s attitudes toward appearance (solid line); however, the relationship of BMI z-scores with teasing was significant for adolescents with high awareness of societal attitudes toward appearance (dashed line).

**Figure 3:**
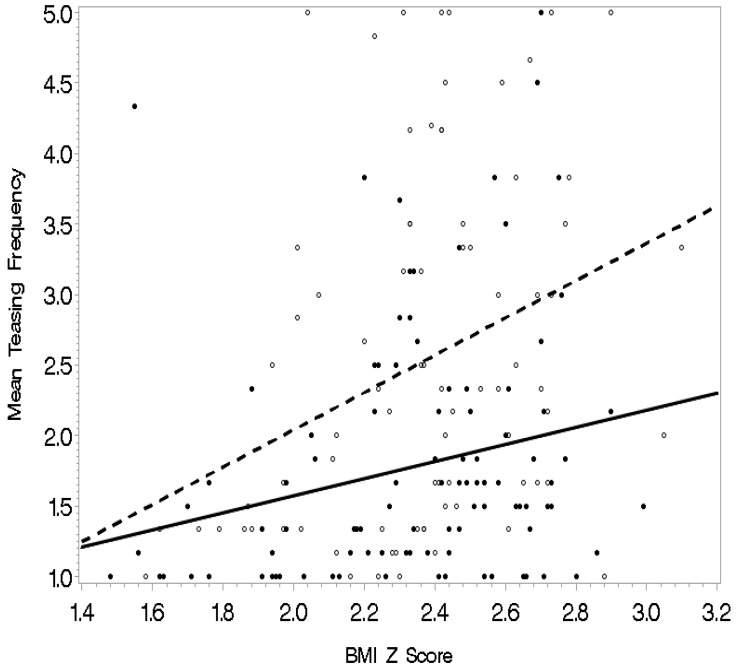
Internalization of society’s attitudes toward appearance as a moderator: The relationship between BMI z-scores and teasing was less strong for adolescents who did not aspire to society’s standards for attractiveness (solid line) as compared to those who internalized those attitudes (dashed line).

**Figure 4:**
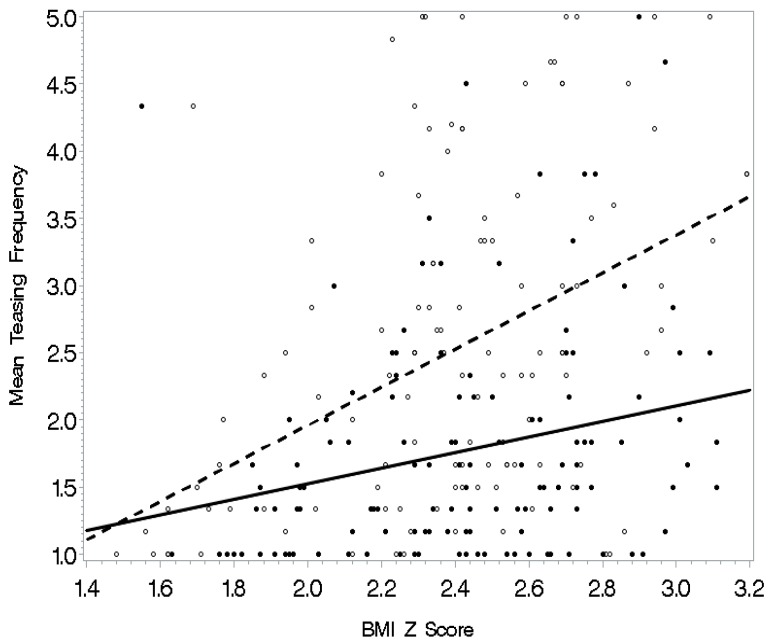
Self-esteem as a moderator: For adolescents with higher self-esteem (solid line), there was less of a relationship between BMI z-score and teasing; this relationship was stronger for those with lower self-esteem (dashed line).

See Table 4 for regression analysis models with interactions predicting frequency of weight-related teasing. Separate multiple regressions were conducted to predict weight-related teasing frequency with each of the societal, physical activity self-efficacy, and psychological functioning variables entered as an interaction term with BMI z-scores as the final step. Body image and BMI z-scores yielded a significant interaction in the prediction of teasing frequency (*p* < 0.05) (see Figure 1). Specifically, BMI z-scores related to mean teasing frequency for adolescents with a more negative body image; the relationship was not evident when body image was positive. Also, the model was significant for awareness of sociocultural attitudes toward appearance with a significant interaction of awareness and BMI z-scores (*p* < 0.01). The relationship of BMI z-scores with teasing was significant for adolescents with high awareness of societal attitudes toward appearance (see Figure 2). For those who did not concern themselves as much with how society defines attractiveness, this relationship was weaker. Similarly, internalization of sociocultural attitudes toward appearance and BMI z-scores yielded a significant interaction in the prediction of teasing frequency (*p* < 0.01). In this interaction (see Figure 3), the relationship between BMI z-score and teasing was apparent for adolescents who were high on internalization of our society’s attitudes toward appearance. The relationship was weaker for adolescents who did not aspire to society’s standards for attractiveness. Finally, the model with the interaction factor of self-esteem and BMI z-scores (*p* < 0.05) indicated that BMI z-scores related to teasing frequency among adolescents with low self-esteem (see Figure 4). For those with high self-esteem, their BMI z-scores related less to being teased.

## 4. Discussion

The major question of this study was to identify and test novel societal and cognitive factors– including society’s role in adolescents’ views on their own attractiveness as well as physical activity self-efficacy—associated with weight-related teasing frequency among treatment-seeking adolescents with obesity. Even after controlling for demographics and BMI z-scores, a cognitive measure that served as a resilience factor was greater physical activity self-efficacy. Adolescents who were determined and confident that they could overcome barriers and be physically active reported less frequent weight-related teasing. This is consistent with Losekam and colleagues’ finding of higher physical activity self-efficacy relating to less weight-related teasing in a German sample of primarily normal weight students in 5th–8th grades [23]. Based on our finding and this previous research [23], physical activity self-efficacy is a reasonable specific target that might lead to less teasing and concomitant improvements in psychological well-being as well as to higher physical activity levels, contributing to weight loss and better conditioning/physical health. More research is needed to empirically test this possibility. 

With the proliferation of social media access among adolescents with greater opportunity for appearance comparisons and awareness of society’s views on attractiveness, investigating how these societal factors relate to weight-related teasing among adolescents who are obese is particularly timely. As the available validated measure in this area was developed for females only, the investigation of these constructs in the current study was limited in scope [15]. In answer to the question of the role of society in weight-related teasing frequency, the interaction models provided significant insights. For those young women who were more aware of societal views on attractiveness and who internalized those views, their degree of obesity mattered more with regard to being teased for their weight. However, for those who were either less aware or who scored lower on internalization of society’s views on attractiveness, their weight status did not factor as much into how frequently they were teased. Perhaps either having less access to judgmental messages or being capable of body acceptance regardless of society’s negative messages offers some sort of protection. Further research into this area is needed. Lending credence to this interpretation is that in the combined sample of males and females, body image and self-esteem also were found to serve as moderators with BMI z-score in predicting weight-related teasing frequency. In all of these cases, the relationship between body composition and teasing mattered least for adolescents who reported positive thoughts about themselves, their bodies, and their place in society. Although a different societal construct, our findings are consistent with those on weight bias internalization relating to weight-based teasing from peers in a similar population [14]. This construct focuses on how adolescents internalize negative stereotypes of being overweight or obese. As Puhl and colleagues noted, additional research in this area is warranted. For addressing internalization of sociocultural attitudes toward attractiveness and weight bias, promising body image program messages that have been rated highly by adolescent girls include the following elements: (1) media images are not real, (2) the ideal body changes over time, (3) the ideal body is different across cultures, and (4) frequently remind yourself to not compare your body to others’ bodies, i.e., avoid the comparison trap [32]. Such body image messages should be tested for adolescents who are obese and also for males to determine if they are rated highly and improve body image and the negative ramifications of internalization of societal attitudes towards appearance.

One of the psychological factors identified as a significant main effect in the prediction of weight-related teasing frequency was depressive symptoms. As had been hypothesized, after taking into consideration demographics and weight status, those adolescents with fewer depressive symptoms reported being teased less often. This finding replicates what has been found previously [4,7,8] and adds to the urgent need to screen and treat elevated symptoms of depression in this population. This lends further support for public health efforts for identification and treatment of depression among our youth.

Of the body composition measures, BMI z-score was the most robust predictor of weight-related teasing frequency. As expected, lower BMI z-scores related to less frequent teasing even within this population of adolescents who were primarily obese. This finding confirms previous research linking obesity to a higher perceived frequency of teasing, independent of demographic covariates [13]. In addition, our study extended the research with the finding that measures of adiposity, percent body fat and fat mass, in addition to BMI z-scores related to teasing frequency in controlled models. 

In terms of demographic factors, the most consistent correlate of weight-related teasing was parental education. Adolescents with parents who had more education reported less weight-related teasing, which has been found in previous research [33]. Additionally, in some models, being male was a protective factor for weight-related teasing. Also, adolescents of non-White race/ethnicity reported less teasing, which is contrary to the finding of no racial/ethnic differences in teasing in a sample of 4746 adolescents across the weight spectrum [6]. Perhaps this different racial/ethnic finding was due to our sample being comprised of adolescents who were primarily obese with the thin-body ideal being less prevalent among African-American compared to White adolescent females [34]. Finally, within our restricted range (13–18 years), age was not a significant covariate in any of the models.

The strengths of this study were a large sample of adolescents who were primarily obese and were well-characterized by multiple objective and self-report measures. Additionally, this sample was diverse in terms of race/ethnicity with approximately half White and half non-White and of socio-economic status with almost a fourth of participants having parents with a high school degree or less of education. Limitations are the cross-sectional nature of the study that does not allow for any causal inferences and the geographic location at one site, limiting generalizability of findings to other regions of the country or to other countries. Additionally, the low internal consistency of the body image measure for this sample should be considered when interpreting those findings; despite this, however, there were significant relationships with weight-related teasing in the hypothesized direction. We also acknowledge the limitation that perceptions about society’s attitudes toward appearance were assessed only for females. This was due to the original version of the SATAQ being created and validated only for females. Subsequent versions of this measure have been modified to include males, but validation separately with males has demonstrated varying factor structures from females [16]. Future research would benefit from including these broader measures to include males when examining awareness and internalization of sociocultural attitudes towards appearance. A related limitation is the use of older measures because this sample was taken from a clinical research program that collected data over time. Additionally, some of the older measures (e.g., of self-esteem and depressive symptoms) were selected due to their excellent established reliability and validity. Finally, our findings must be considered in the context of being conducted with a sample of adolescents with obesity who were being evaluated for enrollment in a weight-management treatment program. Some crucial aspects of the youth and their parents may be different for families who are actively seeking treatment. 

## 5. Conclusions

Having a high BMI z-score alone does not by itself mean that adolescents will be teased about their size. Tailored programs should focus on bolstering adolescents’ positive cognitions about themselves and their bodies and encouraging them to overcome barriers to being physically active so that they will become more confident in their athletic abilities. Public health campaigns may be informed by these findings and promote diverse views about what is attractive in our society. Finally, this is yet another study that underscores the urgent need for screening and treatment of clinically elevated depressive symptoms for this high-risk population.

## Figures and Tables

**Table 1:** Properties of the Major Study Variables.

Variables	*N*	*N* (%)	M (SD)	A	Range (Potential)	Range (Actual)
Demographics						
Age (years)	328		14.59 (1.38)			13–18
Sex—Female	334	213 (63.77)				
Race—White	316	160 (50.63)				
Parent Education— > High School	319	234 (73.35)				
Weight Status/Body Composition Measures						
BMI Z—Score	328		2.41 (0.38)			1.07–3.43
BOD POD Body Fat Percentage	260		45.14 (8.63)			8.30–70.90
BOD POD Lean Body Mass (kg)	241		57.77 (14.91)			19.20–168.10
BOD POD Fat Mass (kg)	241		50.24 (21.49)			18.30–152.50
Societal Measures						
Sociocultural Attitude Awareness	201		3.12 (0.93)	0.84	1–5	1–5
Sociocultural Attitude Internalization	202		2.67 (0.99)	0.91	1–5	1–5
Cognitive Measure						
Physical Activity Self-Efficacy	277		2.24 (0.60)	0.85	1–4	1–3.88
Psychological Functioning Measures						
Body Image	305		3.25 (0.95)	0.31	1–6	1.00–5.82
Self-Esteem	277		20.31 (5.32)	0.79	10–50	12–35
Depressive Symptoms	308		14.48 (11.02)	0.91	0–60	0–52
Outcome						
Weight-Related Teasing Frequency	328		2.10 (1.10)	0.93	1–5	1–5

**Table 2:** Bivariate Analyses of Predictors with Frequency of Weight-Related Teasing.

Predictors	
Demographics	
Sex	0.76
Race	0.75
Parent Education	0.008
Age (years)	0.06 (0.30)
Weight Status/Body Composition	
BMI z-Score	0.31 (<0.001)
BOD POD—Body Fat Percentage	0.29 (<0.001)
BOD POD—Lean Body Mass (kg)	0.15 (<0.02)
BOD POD—Fat Mass (kg)	0.29 (<0.001)
Societal Measures	
Sociocultural Attitude Awareness	0.27 (<0.001)
Sociocultural Attitude Internalization	0.35 (<0.001)
Physical Activity Cognitions	
Physical Activity Self-Efficacy	0.22 (<0.001)
Psychological Functioning	
Body Image	−0.44 (<0.001)
Self-Esteem	0.40 (<0.001)
Depressive Symptoms	0.42 (<0.001)

Categorical variables are reported as *p* values, continuous as Spearman’s *ρ* (*p*).

**Table 3:** Regression Analysis Models Predicting Frequency of Weight-Related Teasing.

Variables	Ƅ (SE)	*p*	Cumulative R^2^	*p* for R^2^ Change
Model 1: BMI Z-Score (*N* = 315)				
Race	−0.19 (0.13)	NS	0.0009	NS
Parent Education	−0.27 (0.14)	NS	0.022	<0.01
Sex	0.17 (0.13)	NS	0.023	NS
Age	0.001 (0.04)	NS	0.024	NS
BMI Z-Score	0.94 (0.17)	<0.001	0.110	<0.001
Model 2: BOD POD Body Fat Percentage (*N* = 255)				
Race	−0.05 (0.13)	NS	0	NS
Parent Education	−0.31 (0.15)	<0.05	0.028	<0.01
Sex	−0.04 (0.14)	NS	0.028	NS
Age	0.02 (0.05)	NS	0.030	NS
BOD POD—Body Fat Percentage	0.03 (0.008)	<0.001	0.089	<0.001
Model 3: BOD POD Fat Mass (*N* = 236)				
Race	−0.14 (0.14)	NS	0	NS
Parent Education	−0.38 (0.16)	<0.02	0.036	<0.01
Sex	−0.003 (0.14)	NS	0.037	NS
Age	0.01 (0.05)	NS	0.039	NS
BOD POD—Fat Mass	0.01 (0.003)	<0.01	0.079	<0.002
Model 4: Physical Activity Self-Efficacy (*N* = 267)				
Race	−0.29 (0.14)	<0.05	0.0002	NS
Education	−0.18 (0.15)	NS	0.017	<0.05
Sex	0.35 (0.15)	<0.02	0.018	NS
Age	−0.01 (0.05)	NS	0.018	NS
BMI Z-Score	0.96 (0.19)	<0.001	0.111	<0.001
Physical Activity Self-Efficacy	0.34 (0.11)	<0.002	0.144	<0.002
Model 5: Depressive Symptoms (*N* = 298)				
Race	−0.15 (0.12)	NS	0	NS
Parent Education	−0.23 (0.13)	NS	0.026	<0.01
Sex	0.03 (0.12)	NS	0.027	NS
Age	−0.002 (0.04)	NS	0.028	NS
BMI Z-Score	0.83 (0.16)	<0.001	0.105	<0.001
Depressive Symptoms	0.04 (0.005)	<0.001	0.289	<0.001

**Table 4:** Regression Analysis Models with Interactions Predicting Frequency of Weight-Related Teasing.

Variables	Ƅ (SE)	*p*	Cumulative R^2^	*p* for R^2^ Change
Model 1: Body Image (*N* = 294)				
Race	−0.08 (0.12)	NS	0.001	NS
Education	−0.27 (0.13)	<0.05	0.024	<0.05
Sex	0.02 (0.13)	NS	0.028	NS
Age	−0.02 (0.04)	NS	0.028	NS
BMI Z-Score	1.73 (0.53)	<0.01	0.118	<0.001
Body Image	0.23 (0.35)	NS	0.264	<0.001
BMI × Body Image	−0.29 (0.14)	<0.05	0.273	<0.05
Model 2: Sociocultural Attitudes Towards Appearance—Awareness (*N* = 191 females)				
Race	-0.17 (0.16)	NS	0	NS
Education	−0.15 (0.17)	NS	0.023	<0.05
Age	−0.07 (0.06)	NS	0.027	NS
BMI Z-Score	−1.09 (0.84)	NS	0.117	<0.001
Awareness	−1.24 (0.61)	<0.05	0.189	<0.001
BMI × Awareness	0.67 (0.25)	<0.01	0.218	<0.01
Model 3: Sociocultural Attitudes Towards Appearance—Internalization (*N* = 191 females)				
Race	−0.12 (0.15)	NS	0	NS
Education	−0.18 (0.16)	NS	0.021	<0.05
Age	−0.08 (0.05)	NS	0.025	NS
BMI Z-Score	−0.83(0.67)	NS	0.113	<0.001
Internalization	−1.18 (0.56)	<0.05	0.221	<0.001
BMI × Internalization	0.66 (0.24)	<0.01	0.253	<0.01
Model 4: Self-Esteem (*N* = 234)				
Race	−0.11 (0.12)	NS	0.002	NS
Education	−0.33 (0.14)	<0.05	0.047	<0.001
Sex	0.10 (0.13)	NS	0.048	NS
Age	−0.003 (0.04)	NS	0.048	NS
BMI Z-Score	−0.47(0.59)	NS	0.147	<0.001
Self-Esteem	0.23 (0.35)	NS	0.264	<0.001
BMI × Self-Esteem	0.07 (0.03)	<0.05	0.305	<0.05
